# Awareness and Willingness of Blood Donation Among Medical Students of Nishtar Medical University, Multan, Pakistan: A Cross‐Sectional Descriptive Study

**DOI:** 10.1002/hsr2.73135

**Published:** 2026-09-02

**Authors:** Areeba Zeb, Fatima Iftikhar, Amna Irshad, Muhammad Faiz Shujra, Amna Naseer, Amna Khalid, Elaf Eman, Sumia Fatima, Muhammad Farhan Jamil, Muddassir Khalid, Fred Segawa

**Affiliations:** ^1^ Nishtar Medical University Multan Pakistan; ^2^ Quaid‐e‐Azam Medical College Bahawalpur Pakistan; ^3^ Watim Medical College Islamabad Pakistan; ^4^ Rawalpindi Medical University Rawalpindi Pakistan; ^5^ Makerere University College of Health Sciences Kampala Uganda

**Keywords:** awareness (D001364), blood donation (D000094345), consent forms (D032962), medical (D013337), Pakistan (D010154), students

## Abstract

**Background:**

Blood is a major component of emergency medical care. The blood transfusion plays a crucial role in saving lives and improving the quality of life. This study aims to assess the medical students’ awareness and willingness regarding voluntary blood donation at Nishtar Medical University, Multan, Pakistan.

**Methods:**

This cross‐sectional study used the convenience sampling method to survey medical students at Nishtar Medical University between May and August 2025. A 48‐item self‐administered questionnaire with four sections—demographics, knowledge, attitude and practice—was used. Tests used in the analysis were the chi‐square, independent sample *t*‐test and one‐way ANOVA test.

**Results:**

A survey of 316 medical students showed a gender distribution of 57.3% females and 42.7% males. Good knowledge of voluntary blood donation (VBD) was found in 110 participants, with only 21.8% aware of the recommended donation frequency. Knowledge varied on donation volume (73.4%), process duration (50.3%), minimum age (16.8%), and weight requirements (41.8%). Only 25.3% knew when postpartum mothers could donate. School was the primary information source (58%). Positive attitudes prevailed, with 72.2% displaying good attitudes, regardless of demographics. However, 81.1% had never donated blood, mainly due to health issues (39.9%) or lack of reasons (32.4%).

**Conclusion:**

The sampled medical students had good knowledge and a good attitude towards VBD, but there was a lack of practice in blood donation. Adequate awareness campaigns are required to increase awareness about voluntary blood donation, which is globally and locally an important healthcare subject.

## 1 Introduction

Blood is regarded as the crucial part of the human body that is maintained by the natural process of synthesis, storage, and utilization [1]. It is indeed an integral component used in healthcare administrations to save the lives of patients in critical conditions of blood loss, such as transplantations, traumas, surgeries, hereditary blood disorders, and other complications [2]. Blood cannot be manufactured artificially, despite the exceptional evolution of medical science, and it relies solely on human donation [1]. Resultantly arises the need for blood transfusion, which saves the lives of millions of people suffering from hereditary blood disorders or chronic diseases that necessitate regular blood administration to make survival possible [3]. Blood transfusions play an important role in saving lives and improving the quality of life. A low rate of blood donation can lead to a shortage of blood and blood products, which can have serious repercussions for patients in need. This situation may result in delays or cancellations of medical procedures and jeopardize the quality of patient care. Blood donation is a critical part of healthcare, ensuring that hospitals and healthcare centers maintain a reliable and secure blood reserve [4].

However, the availability of adequate and safe blood has become a major challenge for developing nations [1]. Numerous barriers in these countries tend to increase the gap between blood supply and demand. Voluntary blood donation is a non‐compensatory act in which an individual willingly donates plasma, blood, or cellular components of blood without receiving any form of payment [3]. Blood donation can take various forms, including plasma, platelet, or whole blood donation. Donors can be categorized as voluntary, replacement, or paid. A replacement donor provides blood to a family member, while a paid donor contributes blood in exchange for money or other compensation [5]. Remarkably, a single unit of donated blood can benefit as many as three individuals when its components are separated [2].

According to the World Health Organization (WHO), currently only 62 countries have blood donation systems that depend entirely on voluntary blood donations. The WHO encourages 100% voluntary blood donation as the primary method to maintain a steady and reliable blood supply. According to the WHO, at least 1% of the population should donate blood to meet national demand. However, in countries with better and more advanced healthcare systems, the blood donation requirements are higher. Globally, in 2006, the blood donation rate was less than 1% (only 10 people donated in a population of 1000) in more than 70 countries [6]. According to researchers in the Netherlands who used data from the WHO 2021 Global Status Report on Blood Safety and Availability, the overall blood donation rate of the 171 countries which were included in the report was 16.3 donations per 1000 residents with the lowest mean value in Yeme (0.6 blood donations per 1000) and the highest blood donation rate in Cyprus (51.7 blood donations per 1000) [7]. According to the WHO, on the basis of samples of 1000 people, in high‐income countries the blood donation rate is 31.5 donations; in upper‐middle‐income countries the blood donation rate is 16.4 donations; in lower‐middle‐income countries the blood donation rate is 6.6 donations; and in low‐income countries the blood donation rate is 5.0 donations [8]. According to studies in China, a lack of blood supply in many cities, such as Beijing, Shanghai, and Guangzhou, results in delay or termination of surgeries [9].

According to studies in Pakistan, voluntary blood donation is very unsatisfactory: 70% of blood is donated by replacement donors, 20% by voluntary donors, and 10% by paid donors [10]. In Pakistan, blood donation and transfusion practices are still developing as compared to blood donations and transfusion practices around the world. In Pakistan, there are about 170 public and 450 private blood banks, mostly hospital‐based. However, there is still a shortage of blood even in large cities, with the blood supply being less than 50% of the requirement [11]. The shortage of safe and voluntary blood donations poses a substantial challenge to effective healthcare delivery. Health facilities across the country require over 5 million blood donations annually, yet receive only approximately 2.3 million, with voluntary non‐remunerated donations contributing just about 18% of the total; the remainder largely comes from family replacement donors, raising concerns about both supply sufficiency and safety. Efforts to promote voluntary blood donation are therefore a priority for national health authorities and the World Health Organization to enhance reliable blood reserves and improve patient outcomes [12]. University and medical students represent a particularly important demographic for addressing this gap. As generally healthy and accessible individuals, they can serve as a sustainable source of voluntary donors. Moreover, medical students are future healthcare providers, and fostering a culture of donation among them can have a long‐term impact on blood donation advocacy and public health awareness. However, research indicates that actual donation rates remain low in this group despite adequate knowledge and positive attitudes, highlighting the need to identify barriers and promote interventions tailored to medical student populations [13].

We are conducting this research to investigate the knowledge, attitudes, and practices of blood donation among medical students at Nishtar Medical University, Multan, Pakistan. While previous studies have assessed knowledge in other areas of Pakistan, there is a lack of data from South Punjab, and this study aims to provide updated information, as previous studies are several years old. This will contribute to bridging the knowledge gap about blood donation and encourage students to donate blood in order to meet the growing demand.

## 2 Methodology

### 2.1 Participants

The study population included all medical students enrolled from first to fifth year at Nishtar Medical University, Punjab, Pakistan. Students who did not provide consent, were critically ill, on leave, postgraduate students, mconfidentally challenged, or lacked internet access were excluded. The final sample size, calculated using the Epi Info sample size calculator for a population of 1500, and a margin of error of 5%, was 306. A convenience sampling method was used to recruit participants.

### 2.2 Study Design and Data Collection

This study employed a correlational cross‐sectional design to assess the influence of knowledge and psychological factors on voluntary blood donation. Data were collected over 5 months (April–August 2025) using a pre‐validated, structured, self‐administered questionnaire based on prior research. The questionnaire consisted of sociodemographic items and three main sections: knowledge (28 items), attitude (9 items), and practice (9 items). Knowledge items included basic knowledge (9 items), criteria for voluntary blood donation (14 items), and knowledge of transmissible diseases (5 items). The questionnaire was administered electronically via web‐based platforms such as Instagram, WhatsApp, and Facebook. Participation was voluntary, and informed consent was obtained from all respondents. A self‐administered structured questionnaire was developed by the investigators after an extensive review of the published literature on blood donation awareness, attitudes, willingness, and practices among medical students. The questionnaire comprised sections on participants’ demographic characteristics, awareness, willingness, attitudes, and blood donation practices. The questionnaire was administered in English, which is the official language of medical education in Pakistan. As the questionnaire was developed specifically for this study, no formal psychometric validation, pilot testing, or reliability assessment was performed prior to data collection.

### 2.3 Statistical Analysis

Data normality was assessed using the Kolmogorov‐Smirnov test. Independent sample *t*‐tests, one‐way ANOVA, and chi‐square tests were used to evaluate relationships between dependent variables (knowledge, attitude, and practice) and independent categorical variables. Knowledge scores were categorized as poor (0–14), fair (15–21), and good (22–28). Attitude scores were classified as negative (0–3), neutral (4–6), and positive (7–9), while practice was considered beneficial if the participant had donated at least once. Analyses were performed using SPSS version 27. Categorical variables were expressed as frequencies and proportions, and statistical significance was set at *p* < 0.05 with 95% confidence intervals.

## 3 Results

### 3.1 Sociodemographic Characteristics of the Cross‐Section

The sample was comprised of 316 medical students from Nishtar Medical University who filled out the questionnaire. The response rate was 100%. Females represented 57.3% (181/316) of participants. The largest two groups were third and second levels (46.2% and 22.5%, respectively) (Table 1).

**Table 1:** Sociodemographic characteristics of study participants.

	*n*	Percent
**Gender**		
Female	181	57.3%
Male	135	42.7%
**Age**		
18–19	37	11.7%
20–21	179	56.6%
22–23	84	26.6%
> 24	16	5.1%
**Academic level**		
First level	41	13%
Second level	71	22.5%
Third level	146	46.2%
Fourth level	29	9.2%
Final level	29	9.2%

### 3.2 Knowledge About VBD

**Figure 1:**
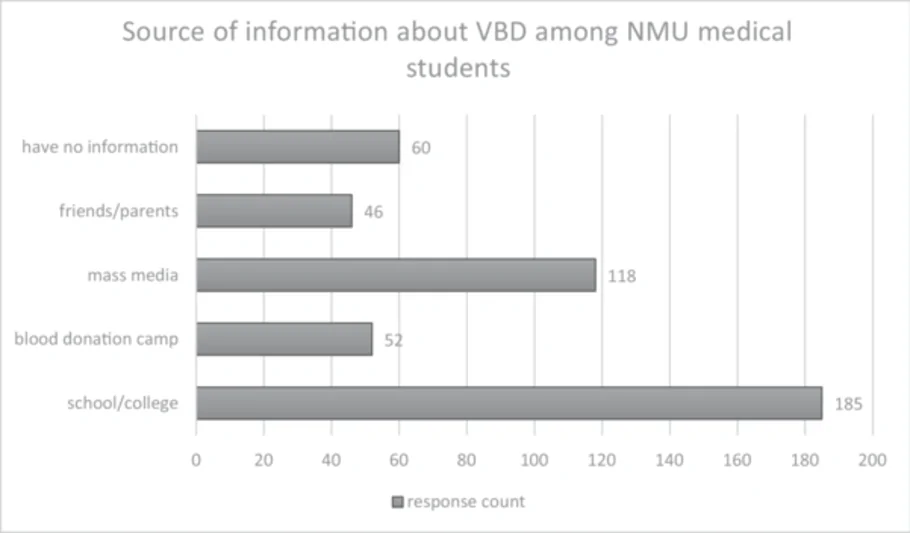
Sources of information regarding voluntary blood donation among medical students at Nishtar Medical University, Multan, Pakistan (*n* = 306). The bar chart shows the number of students reporting each source of information about voluntary blood donation (VBD). The most frequently reported source was educational institutions (school/college; *n* = 185), followed by mass media (*n* = 118), blood donation camps (*n* = 52), and friends or parents (*n* = 46). Sixty participants reported having no prior information about blood donation. Values represent the number of respondents in each category.

Overall, 110 participants out of 316 (34.8%) demonstrated adequate knowledge, 206/316 participants (65.2%) demonstrated fair knowledge about VBD, and 292/316 (92.4%) were familiar with their blood type. Regarding general knowledge about VBD, 69/316 (21.8%) were aware of the healthy frequency for blood donation. 232/316 (73.4%) correctly identified the volume collected during each blood donation, while 159/316 (50.3%) acknowledged the duration of the donation process. Only 53/316 (16.8%) answered the age to start donation correctly, while 132 (41.8%) correctly answered the weight required for donation. 80/316 (25.3%) knew when postpartum mothers can donate blood (Table 2). An independent sample *T*‐test showed no significant difference in knowledge level among genders. There are no significant differences in knowledge levels among age groups and academic levels when tested by the one‐way ANOVA test (Table 3), (Figure 1).

**Table 2:** Participant's response to knowledge about VBD.

	VBD knowledge	Correct answer	*N*	Percent (correctly answered)
General knowledge about blood donation	Do you know your blood group?	Yes	292	92.4%
	How frequently can a person donate?	Every 8 weeks	69	21.8%
	What is the volume of blood collected during each blood donation?	< 500 mL	232	73.4%
	What is the duration of donation process?	20–60 min	159	50.3%
	Is screening blood necessary before blood donation?	Yes	307	97.2%
	Which blood group is considered universal donor?	O‐	272	86.1%
	Which blood group is considered universal recipient?	AB+	287	90.8%
	Can a blood be stored for more than 24 h if not used immediately?	Yes	229	72.5%
	What is blood donation day?	14 June	134	42.4%
Knowledge regarding criteria for a blood donor	What is the minimum age to start blood donation?	16 years	53	16.8%
	What is the minimum weight for blood donation?	55 kgs	132	41.8%
	What is the minimum hemoglobin level required to donate blood?	Male (13) Female (12.5)	125 168	39.6% 7.9%
	What is the required blood pressure at the time of blood donation?	Highest (< 180/100) Lowest (> 90/50)	164 74	51.9% 0.3%
	What is the minimum duration between delivery of baby and blood donation	6 weeks	80	25.3%
	Is fever a contraindication to blood donation?	Yes	210	66.5%
	Are contraceptive pills contraindication to blood donation?	No	140	44.3%
	What minimum interval should be between two successive blood donation processes?	3 months	226	71.5%
	Can all persons with diabetes or hypertension donate?	No	242	76.6%
	Can a pregnant or lactating woman donate?	No	282	89.2%
	Can smokers donate?	Yes	169	53.5%
	Can women donate during menstruation?	Yes	127	40.2%
Transfusion transmissible diseases	Can HIV be transmitted via blood donation?	Yes	293	92.7%
	Can Hepatitis B be transmitted via blood donation?	Yes	294	93%
	Can syphilis can be transmitted via blood donation?	Yes	232	73.4%
	Can Giardia be transmitted via blood donation?	No	149	47.2%
	Can a person be infected by receiving blood transfusion?	Yes	299	94.6%

**Table 3:** Association between KAP components and demographic characteristics.

Demographic characteristics	n	Knowledge score Mean (SD)	*p* value	Attitude score Mean (SD)	*p* value	Practice (Donation History) Percent(Yes)	P^c^‐value
**Gender**							
Male	135	20.71 (1.888)	0.463^t^ 0.634^c^	7.7778 (1.42822)	0.686^t^ 0.110^c^	14.6%	< 0.001
Female	181	20.54 (2.127)		7.8508 (1.69799)		4.1%	
**Age**							
18–19	37	20.64 (2.287)	0.981^a^ 0.981^c^	7.729 (1.574)	0.553^a^ 0.362^c^	5.4%	< 0.001
20–21	179	20.58 (2.032)		7.910 (1.488)		15.1%	
22–23	84	20.67 (1.933)		7.631 (1.822)		27.4%	
> 24	16	20.50 (2.000)		8.000 (1.366)		43.8%	
**Academic level**							
First level	41	20.58 (2.109)	0.703^a^ 0.775^c^	7.682 (1.822)	0.271^a^ 0.417^c^	12.2%	0.003
Second level	71	20.57 (2.162)		7.718 (1.523)		11.3%	
Third level	146	20.53 (1.969)		7.739 (1.673)		17.1%	
Fourth level	29	21.13 (1.865)		8.310 (1.365)		37.9%	
Final level	29	20.62 (2.077)		8.172 (0.966)		34.5%	

Abbreviations: a, one‐way ANOVA test; c, chi‐square test; p_c_, *p* value of chi‐square test; t, independent sample *t*‐test.

### 3.3 Attitudes Towards VBD

Majority of participants showed good attitude, representing 72.2% (228/316). For instance, 98.4% (311/316) believed that blood donation is a noble act. Furthermore, 90.8% (287/316) consider voluntary blood donation as the best source of donation. Regarding willingness to donate, 93.0% (294/316) were willing to donate blood to a relative, 257/316 (81.3%) to anyone, and 287(90.8%) were willing to donate blood regardless of patient's religion. 280/316 (88.6%) did not expect any reward in return (Table 4). There was no significant difference in attitude level among gender, age, and academic levels assessed by independent sample *t* test and One‐ way ANOVA (Table 3).

**Table 4:** Participant's attitude towards VBD.

VBD attitude items	Favorable attitude	Participants	Percent
Is blood donation a good and noble act?	Yes	311	98.4%
What do you think is best source of blood donation?	Voluntary	287	90.8%
What is your attitude towards blood donation?	Agree	260	82.3%
Are you willing to donate to your relative?	Yes	294	93%
Are you willing to donate blood to anyone?	Yes	257	81.3%
Will you donate blood without knowing religion of recipient?	Yes	287	90.8%
Do you expect any reward from blood donation?	No	280	88.6%
Would you like to receive training on blood donation?	Yes	252	79.7%
If your university organizes a donation camping, would you participate and donate?	Yes	243	76.9%

### 3.4 Practice Towards VBD

**Figure 2:**
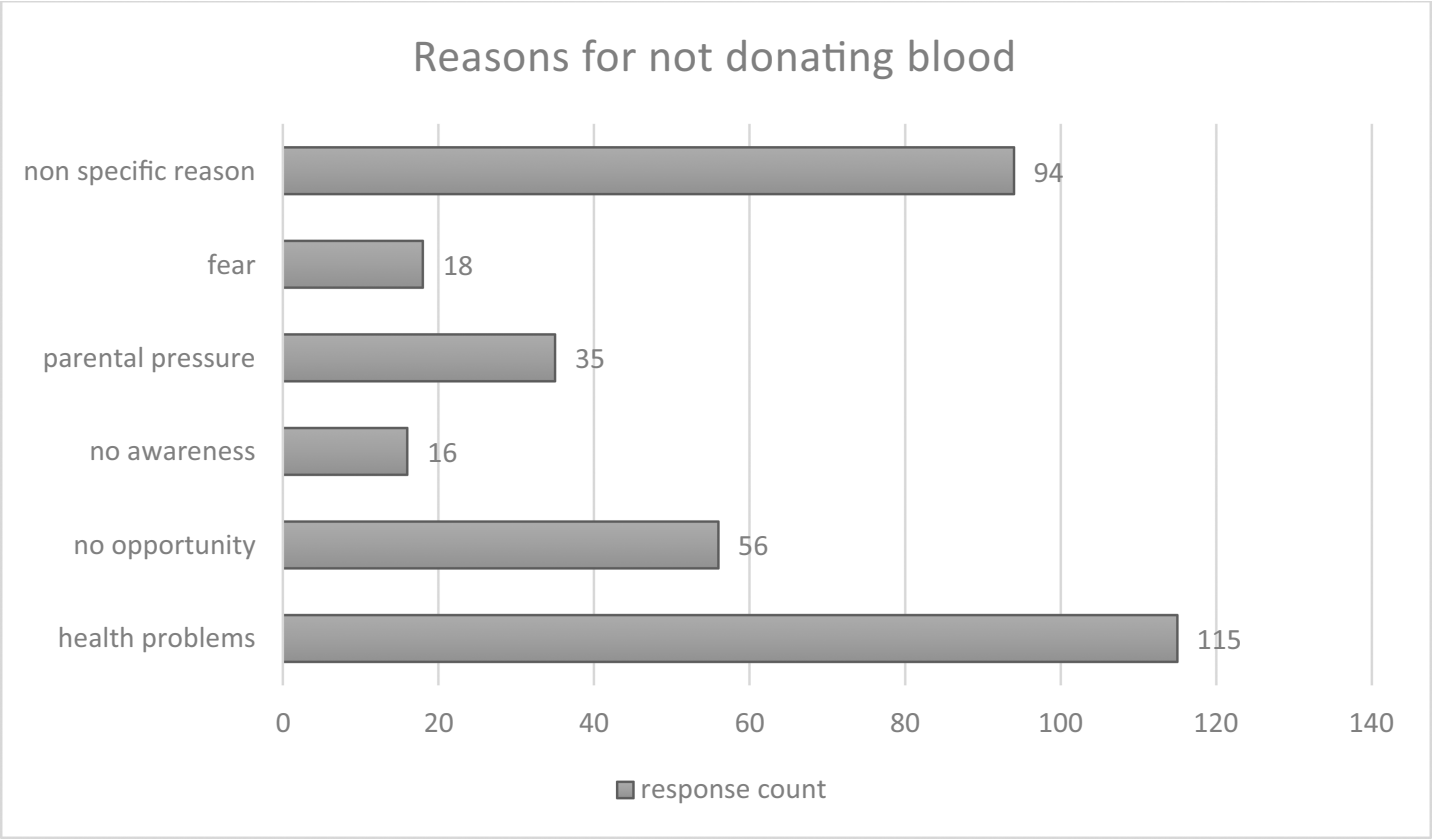
Reasons for not donating blood among non‐donor medical students at Nishtar Medical University, Multan, Pakistan. The bar chart illustrates the number of non‐donor participants reporting each reason for not donating blood. The most commonly cited reason was health problems (*n* = 115), followed by non‐specific reasons (*n* = 94) and lack of opportunity (*n* = 56). Other reported reasons included parental pressure (*n* = 35), fear (*n* = 18), and lack of awareness (*n* = 16). Values represent the number of respondents selecting each category.

Out of 316 participants, 59 (18.7%) had donated blood before, with 16 (5.1%) having donated blood more than once. The majority, 68 (21.5%) had donated blood voluntarily. The two major reasons for not donating blood are health problems, 115/316 (97%), and no opportunity, 56/316 (43%) (Table 5) and (Figure 2). There was a significant association between gender and donation practice (*p* < 0.001), with males more likely to donate blood than females. Academic level of students and donation history were also significantly related (*p* = 0.003) with increased likelihood of donation in level 3 and above. Association between the age of participants and donation practice was also significant (*p* < 0.001), with participants older than 22 more likely to have donated blood than their younger group (Table 3).

**Table 5:** Participant's practice towards VBD.

VBD practice items	Practice	Participants	Percent
Have you donated blood before?	Yes	59	18.7%
What type of blood donation has you practices before?	Paid	5	1.6%
	Replacement	4	1.3%
	Voluntary	68	21.5%
Are you willing to donate blood in future?	Yes	276	87.3%
Have you received blood?	Yes	18	5.7%
Why did you donate blood?	Non relative needed blood	11	3.5%
	Relative needed	17	5.4%
	blood Voluntary	56	17.7%
How often do you donate blood?	Irregularly	43	13.6%
	Regularly more than twice a year	9	2.8%
	Regularly once a year	10	3.2%
	Regularly twice a year	5	1.6%
How many times have you donated blood before?	Once	31	9.8%
	Twice	16	5.1%
	More than twice	14	4.4%

## 4 Discussion

This study assessed the knowledge, attitude, and practice (KAP) of voluntary blood donation among medical students at Nishtar Medical University, Multan. The findings revealed a significant contraindication; however, the majority of students demonstrated a solid understanding and an optimistic outlook towards blood donation. The actual frequency of previous donation remained considerably low. This inconsistency hints at a constant global and local gap between perception and action, even in well‐informed groups like health care professionals.

Our research revealed that 206 students possessed a fair level of knowledge and 110 exhibited good understanding. This comprehensive understanding among medical students is not overwhelming, given their continuous exposure to health sciences and public health education. These outcomes are consistent with other studies conducted among medical students in Qatar, Syria, and Gaza, all of which stated increased knowledge scores regarding blood donation [2, 3, 14]. A similar pattern was observed in India, where undergraduate medical students demonstrated healthy awareness of organ eligibility, perks of blood donation, and risks associated with transfusion‐transmissible infections [15, 16].

Moreover, the overwhelming majority of participants in our research—more than 90%—showed a positive attitude towards blood donation, confirming that it is a dignified act and valuable to society. These favorable attitudes are reflected in studies from Ethiopia [17], Saudi Arabia [18] and even multi‐national surveys like that of [1] Similar findings from [19] reported that students possessed positive attitudes but were unsure about safety, which might influence actual participation. This showed that university students strongly support voluntary donation when presented with hypothetical situations. These results confirm that education campaigns and medical training programs have been effective in developing positive perceptions of blood donation.

Despite these promising levels of knowledge and attitude, a small fraction of students had donated blood, less than 20%. This implies that noble intent has not turned into action, a challenge observed across all KAP studies globally [20, 21]. In Ethiopia [5], noted a similar pattern of low donation rate despite good knowledge and attitude. This idea aligns with [22] demonstrating only 17.3% of Taif students donated blood, although many possessed good knowledge, with only less than half of those knowledgeable donating. This discrepancy suggests that factors beyond knowledge and attitude may be crucial in influencing behavior. Therefore, understanding and dealing with these barriers is essential.

In our research, several barriers to blood donation were identified. The most frequently mentioned were health issues (39.5%), unspecified reasons (32.2%), and lack of opportunity (19.2%). Additionally, psychological and social factors such as fear (6.2%), parental pressure (12%), and lack of awareness (5.5%) were also noted. These challenges are not exclusive to our study. Studies conducted in Gaza and Ethiopia reported similar limitations preventing voluntary blood donation among students [3, 17, 23]. Also stated low practice in Tanzania due to fear, myths, and logistic inefficiencies. This recurring issue accentuates the need for the development of better structural and emotional support systems to help transform intent into action. In contrast, a survey from Khulna City [24] revealed a 64.7% voluntary donation rate despite 68.6% of knowledge level and 98.3% positive attitude.

Statistical analysis indicates a significant correlation between demographics and blood donation history. Male students were more inclined to donate blood, as proven by other large‐scale studies such as [1], which stated a similar gender contrast among 16 countries [21]. Also reported increased donation rates among male students in Ethiopia. The Harari study [25] Reported an odds adjustment ratio of 4.17 for males and health science students. Aligning with our findings [26] Reported that males donated more, with fear and logistical issues being major challenges for female donation. Sociocultural elements may contribute, including societal standards that deter females from donating blood due to possible risks associated with health, menstruation, and familial resistance.

Similarly, academic level was a strong predictor of blood donation. Students in their third year and beyond donated blood more frequently. This is presumably due to increased clinical exposure and medical awareness of blood transfusion as they proceed through their medical education. Studies from Ethiopia and India support this analysis, where senior medical students were more likely to practice health‐promoting activities [15, 17].

Age also had a significant affiliation with practice. Students over the age of 22 were more likely to have donated blood. Older age is often associated with maturity, a sense of civic responsibility, and independent decision‐making capacity [27] and [21] revealed similar age‐related tendencies in their respective studies in India and Ethiopia [28]. Reported that older nursing students had increased knowledge and attitude levels, mirroring our finding that students older than 22 donated more. Likewise, study from [24] quoted that male students, older age, and higher academic levels are more likely to donate. These demographic perceptions are necessary for designing focused strategies.

Regarding information source, most of our students specified school or college as the main source of information concerning blood donation, followed by mass media. Friends, family, and public blood camps were also important sources. While it highlights the positive role of academics, the relatively small impact of public campaigns and media demonstrates the underutilization of an important communication tool that could improve outcomes.

Worldwide, blood shortage remains a serious public health concern. A 2025 report by NHS Blood and Transplant England issued an urgent call for one million new donors, revealing a significant shortfall of over 200,000 donations annually [29] This underscores the necessity of proactive blood donor induction not only in poor nations but also in high‐income countries. In contrast, Pakistan's blood donation system is strongly reliant on replacement donors, with voluntary donors comprising only 20% [11, 20]. This fact accentuates the urgency for reform in economically challenged countries.

Health organizations hold hidden ability in modifying donation outlook into conduct. Given that medical students already possess beneficial insight and attitude scores, focused behavioral mediation can drastically improve practice. This may include incorporating donation‐related activities into the academic schedule, hosting on‐campus blood donation camps, and providing certificates, public acknowledgement, or academic credits as formal rewards.

## 5 Limitations

This research has various limitations. Being confined to a single medical institute in South Punjab, findings may not apply to other institutions or regions. The cross‐sectional design of the study prevents evaluation of trends over time or establishment of causality. Additionally, reliance on self‐reported data may introduce the possibility of response bias, either due to social recognition or misinterpretation of questions. Further studies should review multi‐center schemes with longitudinal follow‐up to better comprehend the transition of knowledge and attitude into practice. Furthermore, the questionnaire was developed specifically for this study and did not undergo formal psychometric validation, pilot testing, or reliability assessment. Therefore, although it was developed based on an extensive review of the literature, measurement error cannot be completely excluded.

Regardless of these limitations, findings provide a clear direction. Medical universities must adopt multi‐level interrelationships that merge knowledge, opportunity, and motivation. Campaigns that deal with exclusive barriers of fear, health misconceptions, or opportunistic deficits should be prioritized. Moreover, integration of blood awareness in academic programs, especially during early years, would help bridge the gap between knowledge and practice, even before clinical training begins.

## 6 Conclusion

In conclusion, this study underlines the persistent gap between knowledge and practice in relation to blood donation. While awareness and attitudes remain encouraging, actual practice remains low. Identification of principal demographic and psychological barriers in this study offers a valuable contribution to designing effective strategies. Alignment of student‐centered strategies with global causes like the NHS and WHO may help ensure a secure, voluntary, and renewable blood supply.

## 7 Future Implications

This study highlights the need for strategies that can convert favorable knowledge and attitudes toward blood donation into consistent practice. Future initiatives should focus on addressing key barriers such as health concerns, fear, and limited opportunities. On‐campus donation programs and incentives for participation should also be promoted. Incorporating blood donation awareness and activities into medical curricula, alongside targeted media and peer‐led campaigns, may further enhance engagement. Additionally, multi‐center and longitudinal studies must be done to assess the effectiveness of these interventions across different sociocultural settings, providing evidence‐based guidance for policies that ensure a sustainable and voluntary blood donor pool.

## Author Contributions

AZ, FI, AI, MF, SAN, AK, EE and MFJ contributed to the literature search, study screening, data extraction, data analysis, interpretation of findings, and manuscript preparation. MK provided supervision, project administration, final review, and editing of the manuscript. SF provided supervision, critical review, and final approval of the manuscript. FS provided resources. All authors read and approved the final version of the manuscript.

## Funding

The authors have nothing to report.

## Ethics Statement

In this study, all methods were carried out in accordance with relevant guidelines and regulations. The study adhered to the Declaration of Helsinki in this regard. The current research was done after obtaining the approval of the Ethics Committee of Nishtar Medical University, Multan, Pakistan (Ethical code: 8972/NMU) on April 26, 2025.

## Conflicts of Interest

The authors declare no conflicts of interest, financial or otherwise, that could have influenced the research, affirming that this study was conducted independently and without any commercial or financial relationships that could be perceived as a potential conflict of interest.

## Transparency Statement

The corresponding author affirms that this manuscript is an honest, accurate, and transparent account of the study being reported; that no important aspects of the study have been omitted; and that any discrepancies from the study as originally planned have been explained.

## Data Availability

Data that support the results of this study are available from the corresponding author upon request.
